# Adipose-Derived Mesenchymal Stem Cells in the Use of Cartilage Tissue Engineering: The Need for a Rapid Isolation Procedure

**DOI:** 10.1155/2018/8947548

**Published:** 2018-04-03

**Authors:** Sam L. Francis, Serena Duchi, Carmine Onofrillo, Claudia Di Bella, Peter F. M. Choong

**Affiliations:** ^1^Department of Surgery, The University of Melbourne, Melbourne, VIC, Australia; ^2^Department of Orthopaedics, St. Vincent's Hospital, Melbourne, VIC, Australia

## Abstract

Mesenchymal stem cells (MSCs) have shown much promise with respect to their use in cartilage tissue engineering. MSCs can be obtained from many different tissue sources. Among these, adipose tissue can provide an abundant source of adipose-derived mesenchymal stem cells (ADMSCs). The infrapatellar fat pad (IFP) is a promising source of ADMSCs with respect to producing a cartilage lineage. Cell isolation protocols to date are time-consuming and follow conservative approaches that rely on a long incubation period of 24–48 hours. The different types of ADMSC isolation techniques used for cartilage repair will be reviewed and compared with the view of developing a rapid one-step isolation protocol that can be applied in the context of a surgical procedure.

## 1. Introduction

Cartilage tissue engineering has become a major research interest in the past few decades, primarily due to the inability of native human cartilage to self-repair [1, 2]. There is no reliable long-term joint preserving management option for early onset arthritis secondary to cartilage defects, and this may potentially lead to joint replacement (arthroplasty) and associated short- and long-term risks and sequelae [3, 4]. Fibrocartilage formation is the major barrier in the long-term viability of currently used clinical methods and is detrimental to joint function [5, 6].

The diamond concept [7] embodies the 4 major strategies that underpin tissue engineering, namely, cells, scaffolds, growth factor/cytokines, and environmental stimulation.

This review will focus specifically on ADMSC isolation techniques and their efficiency with respect to driving cartilage formation.

Current isolation procedures in cartilage tissue engineering are in vitro and laboratory-based. These are primarily complex two-step procedures that also raise ethical concerns with respect to human tissue culture in a laboratory setting [8].

Translating these techniques into the clinical setting will require the development of a rapid, sterile, one-step technique that could fit into a day surgery timeframe. To date, rapid isolation of bone marrow-derived MSCs [9, 10] and their therapeutical potential has been studied [11], but an important barrier to adoption has been the low number of stem cells requiring a period of cell expansion in the laboratory. There is only one published study assessing a rapid isolation protocol (<30 minutes) for ADMSCs from abdominal lipoaspirate [12], but even this technique relies on a minimum of 24 hours for plastic adherence.

## 2. Adipose-Derived Mesenchymal Stem Cells

ADMSCs have the ability to differentiate into mesodermal tissue lineages, that is, bone, cartilage, muscle, and adipose [6, 13–16]. They have been incorporated into many different scaffold-based systems and have an established role in cartilage tissue engineering [17, 18].

Initially, bone marrow (BM) was the most commonly used source of MSCs. Like ADMSCs, BM-derived MSCs are multipotent in nature and can produce tissue of mesodermal lineage [19]. Tissue can be harvested autologously and does not pose the ethical, tumorigenic, or immunogenic risk as presented by pluripotent stem cells. The disadvantages of using BM include low tissue volume and low cell volume [13, 20, 21]. BM-derived MSCs are comparable [22], if not inferior, in respect to chondrogenic potential when compared to ADMSCs [22, 23]. These factors, in addition to less invasive tissue harvesting techniques, make adipose tissue a more desirable source.

## 3. Tissue Sources and Harvesting Techniques

ADMSCs can be obtained from different sources and by different techniques. The two major sources are abdominal fat and infrapatellar fat pad (IFP). Techniques and protocols for ADMSC harvest and isolation vary based on different laboratory groups. Abdominal fat can be harvested from subcutaneous tissue via abdominoplasty or arthroscopy.

**Figure 1:**
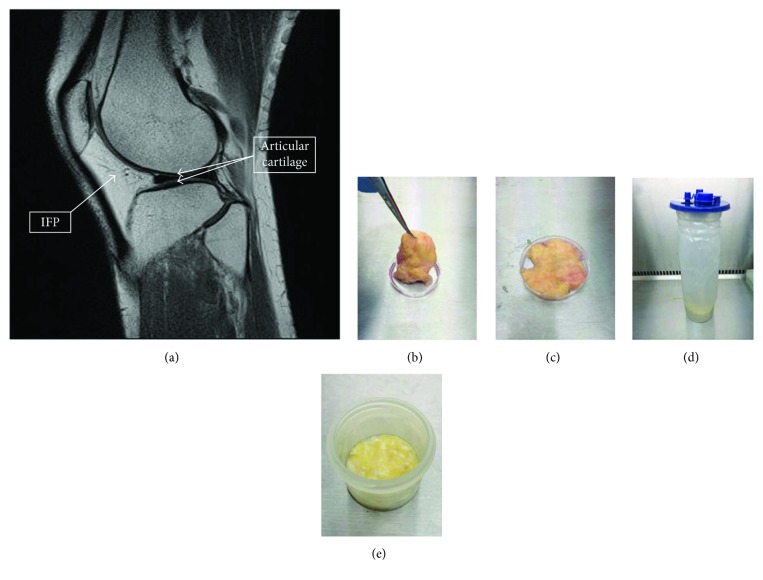
(Modified and used with permission from Wiley under CC BL). Infrapatellar fat pad (IFP) location and harvested tissue. (a) Sagittal magnetic resonance imaging scan of the knee showing the relationship of the IFP (arrow) to the articular cartilage (double arrow). (b, c) Excised IFP from a patient undergoing knee arthroplasty (b) has the fat removed from the fibrous tissue (c). (d, e) The arthroscopically harvested fat pad (d) was separated from the irrigation fluid before enzymatic digestion (e).

The IFP (Figure 1(a)) is an emerging source of MSCs for cartilage tissue engineering [24, 25]. IFP can be opportunistically harvested (Figure 1(a)) during routine surgical procedures such as knee arthroplasty (Figures 1(b) and 1(c)) or arthroscopy (Figures 1(d) and 1(e)) and is known to have high chondrogenic potential [26]. Although there is less fat volume in the IFP compared to abdominal fat, chondrogenic potential has been shown to higher in ADMSCs sourced from the IFP [27, 28]. The proximity of the IFP to the knee joint may account for this higher potential.

These results could pave the way for future novel advances in minimally invasive arthroscopy or techniques for pure fat pad harvesting as opposed to opportunistic harvest and, better yet, the possible establishment of a single-step surgical repair technique using stem cell technology.

## 4. Cell Isolation Procedure

Obtaining a stem cell population requires several sequential steps, including harvest, mechanical breakdown, chemical breakdown, purification, and plastic adherence. After these steps, it is important to count and characterise cells and their stemness potential with appropriate investigations.

Cell expansion plays a crucial role to allow adequate cell numbers required for in vitro studies. However, when considering an in situ one-step regenerative procedure for chondral defects, initial cell harvest numbers will need to be adequate for repairing variably sized lesions. Approximately one million cells are needed for a 1 cm^3^ lesion [29]. Therefore, studies into cell numbers per tissue unit harvested will be crucial. Recently, cell aggregates have demonstrated increased proliferative ability. This may be due to direct cell-cell contact, allowing better intracellular communication [30–32]. It will be important to now study the number of aggregated cells needed to repair variably sized lesions; if less than one million cells are needed per 1 cm^3^ lesion, this could prove to be a major breakthrough.

**Figure 2:**
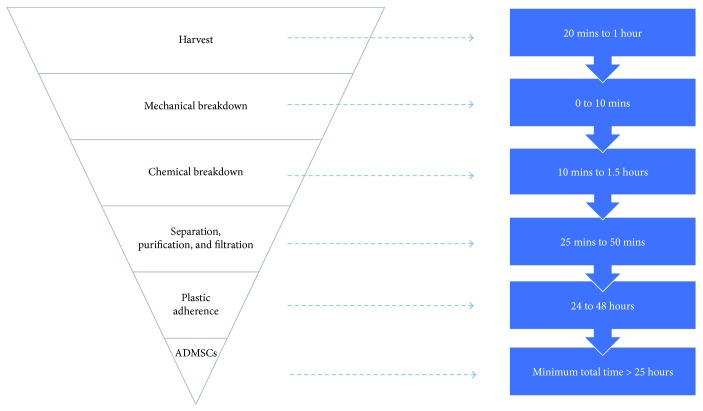
Adipose-derived mesenchymal stem cell (ADMSC) isolation protocol including timeframes.

The steps involved and respective timeframes when using standard protocols are shown below (Figure 2). With both sources, current techniques take >1 hour for cell isolation and subsequently require incubation for up to 24–48 hours to allow for plastic adherence [33]. This was proven to be a lengthy procedure which is not a major concern if only applied to in vitro studies.

### 4.1. Harvest

Abdominal fat can be harvested endoscopically or via abdominoplasty with no significant difference in cell structure and the number of cells yielded per unit of volume [34]. Both take minimal procedural time of <20 minutes; however, the intended abdominoplasty procedure may take much longer. Minimal comparisons are present in the literature. Two studies showed the comparable morphology of cells harvested from endoscopic (liposuction) and abdominoplasty (resection) techniques; however, inadequate phenotyping and characterisation of isolated cells were undertaken in both studies [35–37].

Infrapatellar fat pad (IFP) can be harvested via arthroscopy and opportunistically from arthroplasty. While both tissue harvesting techniques only require the minimal procedural time of <20 minutes, the overall arthroplasty procedure may take up to 2 hours. Additionally, isolated ADMSCs from IFP have been shown to have higher chondrogenic potential compared to cells isolated from bone marrow [26] and abdominal adipose tissue [27], making them a superior source.

### 4.2. Mechanical Breakdown

Abdominal fat harvested via liposuction is obtained in lipoaspirate form and does not require any further mechanical breakdown. IFP tissue requires separation of fat from the fibrous pad via a scalpel which takes roughly 10 minutes [38].

### 4.3. Chemical Breakdown

Once fat particles have been isolated from both sources, collagenase is added to the samples to allow chemical breakdown of the tissue. While a number of collagenases are available for ADMSC isolation (Table 1), type 1 collagenase is the preferred agent for isolation prior to chondrogenic lineage induction. Research shown using collagenase type 1 at 0.2% for 10 minutes of chemical/enzymatic breakdown can obtain a stromal vascular fraction [39]. Increased time > 30 minutes using collagenase digestion has been shown to reduce the number of viable adipocytes [40]. Adding trypsin to pure collagenase allows for maximal digestion [41]. Further study is warranted to find the optimal type and concentration of collagenase to enable rapid, effective, and efficient disaggregation of AMDSCs.

After the addition of collagenase, the samples are incubated and agitated with a rotating platform (≥100 revolutions per minute). During incubation on the platform, both chemical and mild mechanical agitations occur synergistically.

### 4.4. Purification

Purification refers to the separation of material, the removal of unrequired product, and filtration. Following mechanical and chemical breakdowns, the sample undergoes a universal step of centrifugation for 10 minutes [34, 38]. Next, the supernatant is removed, and the pellet is washed with phosphate buffer solution before being filtered through a sterile 100 *μ*m filter. After another round of centrifugation for 5 minutes, the supernatant is discarded and the remaining pellet is then resuspended in 5 millilitres of red cell lysis buffer [28] for 10 minutes before being filtered through a sterile 40 *μ*m filter [38]. After a final 5-minute round of centrifugation, the resulting supernatant is removed, leaving a cell pellet. This total purification procedure is reported to take anywhere between 25 and 50 minutes. Postpurification cells are resuspended in culture media and then counted prior to being plated in flasks.

### 4.5. Plastic Adherence

Once cells are plated in appropriate flasks based on cell counts, they are incubated usually for 24–48 hours. Cell attachment to plastic is a key step for identifying and isolating cells with stem cell characteristics. Unattached cells are discarded. At this stage, the attached cells can be expanded and passaged or frozen in liquid nitrogen for later use. This plastic adherence step requires a minimum of 24 hours of incubation. Cell sorting using marker selection (flow sorting) is an alternative to plastic adherence with respect to isolating a pure ADMSC population [57]. The drawback to this technique is the time requirement and lack of exact phenotypic characterisation of ADMSCs.

### 4.6. Phenotype

As per the International Society for Cellular Therapy [58, 59], three criteria must be fulfilled for the MSC phenotype: adherence to plastic, appropriate surface antigens, and expression of multipotent differentiation potential.

**Figure 3:**
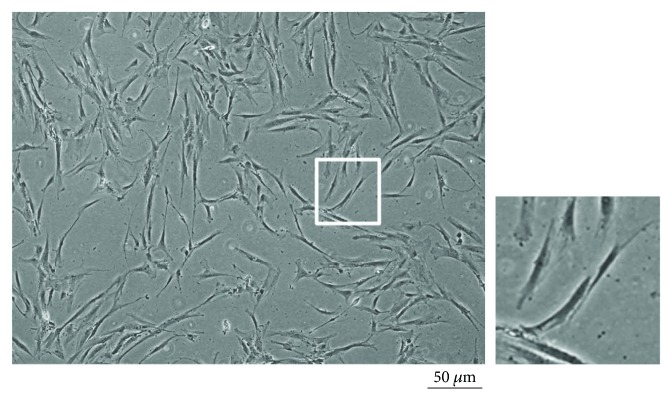
Plastic adherence and morphology of mesenchymal stem cells isolated from the infrapatellar fat pad, representative view using bright field microscopy.

Plastic adherence is a hallmark property of all MSC groups [60, 61]. Furthermore, typical morphology and colony formation can be observed under a microscope as seen in Figure 3.

To confirm the phenotype of cells isolated as MSCs, specific surface antigens are tested through immunophenotyping and can be done via flow cytometry [46]. MSCs generally express (≥95%) CD73, CD90, and CD105, while lacking expression (≤2%) of CD 11b, CD14, CD34, CD45, and CD79a [47].

However, exact characterisation is still in development [57, 62], and surface phenotyping should be used in conjunction with other criteria to help best identify MSC.

Biologically, MSCs should display three lines of differentiation potential: osteoblasts, adipocytes, and chondroblasts [58]. Multipotent potential can be evidenced by differentiation into various lineages using different induction paths [16] and can be tested with staining and qPCR (Table 2).

## 5. Rapid Isolation Procedures in Literature

Over the past decade, several commercially available enzymatic and nonenzymatic adipose tissue cell isolation systems [63, 64] have achieved sterile processing and high yields of cells. However, these systems only isolate a stromal vascular fraction (SVF), implying that a plastic adherence step is still required for pure ADMSC isolation.

One published attempt at a rapid protocol using abdominal lipoaspirates achieved an SVF isolation within 30 minutes [12]. 2.5 × 10^5^ ADMSCs were isolated using the 30-minute approach compared to 2.0 × 10^6^ from the standard approach. The final step of plastic adherence to isolate a pure ADMSC population still required 24–48 hours of further incubation. Furthermore, the number of ADMSCs yielded was nearly 10 times less when compared to the standard procedure.

A purely nonenzymatic breakdown approach with blender mixing and sonication has been used to obtain an SVF within 25 minutes [65]. On average, 2.6 × 10^5^ cells were isolated in the SVF, resulting in a very low average of 2.4 × 10^4^ ADMSCs. Although SVF isolation is rapid, overnight (>24 hours) plastic adherence is once again still required to obtain a pure ADMSC population.

It is evident from these two approaches that a low number of cells are obtained, possibly due to toxicity from the methodology. Moreover, only an SVF was rapidly isolated as opposed to a pure ADMSC population, which still takes >24 hours.

The use of SVF alone, without the use of a pure ADMSC population, may be another therapeutic option. As mentioned earlier, given the superior chondrogenic potential of ADMSCs isolated from the IFP [27, 28], SVF populations from abdominal fat should be compared to IFP before trialling SVF as a direct one-step therapeutic option. However, the lack of cell-cell contact within an SVF due to scattered ADMSCs will lead to inferior cartilage repair as a result of reduced paracrine stimulation [30].

## 6. Where Can We Save Time?

**Figure 4:**
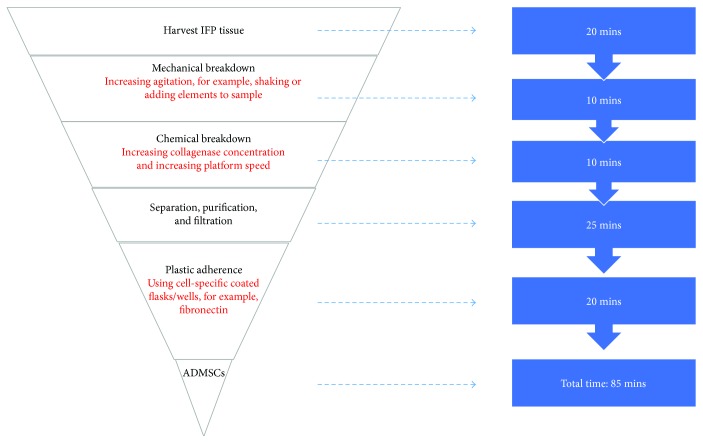
Proposed rapid adipose-derived mesenchymal stem cell (ADMSC) isolation procedure from the infrapatellar fat pad (IFP). The three major changes proposed are highlighted in red.

There are three procedural steps where time could be saved. These are discussed below and also represented in Figure 4.

### 6.1. Mechanical Breakdown

The initial mechanical breakdown could be further enhanced by adding mechanical agitation through shaking, vortexing, and possibly adding sterile solid materials during chemical breakdown to synergistically assist the breakdown of tissue. Sterile beads have been used commercially in liposuction kits to help emulsify tissue [66]. If such materials were to be used, they need to be sterilisable and nontoxic and show a consistent and predictable effect on tissue breakdown based on morphology and weight. These factors will need proper investigation prior incorporation into isolation techniques. The risk of these more vigorous approaches is cell damage and death; therefore, it will be important to assess cell viability in such intended studies.

Although it currently only takes 0–10 minutes to break tissue down depending on the source, the more vigorous breakdown of tissue earlier, particularly of IFP tissue, may help reduce the total time needed for the subsequent step of chemical breakdown.

### 6.2. Chemical Breakdown

The chemical breakdown shows varying timeframes with reports of 10 minutes for the breakdown of tissue into an SVF [39]. Higher concentrations of collagenase with the addition of trypsin may allow for the maximal breakdown, while higher rpm use on rotating platforms may enable synergistic breakdown. Once again, the possibility of cell toxicity will need to be investigated [40].

### 6.3. Plastic Adherence

Plastic adherence forms the major time barrier (minimum of 24 hours). A new rapid technique needs to be established in this step as this timeframe is not clinically feasible.

Recent literature has reported the high affinity of articular progenitor cells (APCs) to fibronectin, with research showing APC adherence to fibronectin-coated wells in 20 minutes [67]. Although lacking clearly defined markers, these APCs, also known as chondrogenic progenitor cells, have shown stem cell potential and are similar to and possibly more differentiated forms of ADMSCs [68].

This is a major finding supporting the use of fibronectin-coated wells or plates to isolate ADMSCs if it can be demonstrated through immunophenotyping that stem cells are attaching selectively to the coating.

## 7. Conclusion and Future Clinical Applicability

A rapid ADMSC isolation technique is necessary for a single-step, tissue engineering-based surgical repair of cartilage tissue. Literature to date suggests IFP-harvested ADMSCs to be the most promising in chondrogenic potential. If a procedure can isolate ADMSCs using an approach such as that described in Figure 4, then incorporating the cells into a matrix and implanting them into a defect using handheld bioprinters [69–71] may pave the way for a single-step intraoperative cartilage repair technique. When leveraging the advantages of a day-only minimal incision surgery, such as arthroscopy, there may be significant clinical outcome and health care cost gains.

The future of cartilage repair is promising. By speeding up cell isolation techniques, a major time barrier can be overcome, translating a clinically to a nonlaboratory-based procedure, shorter surgical time, quicker recovery for the patient, and a smaller burden on the health care system. Younger patients can now hope for a simple, low-risk treatment option that aids in preventing the onset of osteoarthritis.

## Figures and Tables

**Table 1:** Comparison of human studies using enzymatic breakdown with collagenase for ADMSC isolation from subcutaneous tissue. Phosphate buffer solution (PBS), Hank's balanced salt solution (HBSS), bovine serum albumin (BSA), and Dulbecco's modified eagle's medium (DMEM).

Author	Collagenase	Concentration	Dilution media	Enzymatic duration
Cheng et al. [42]	Type 1	0.1%	PBS	60 minutes
Choudhery et al. [43]	Type 4	0.2%	PBS	20 minutes
Satish et al. [44]	Type 2	0.1%	HBSS/BSA	40 minutes
Kinoshita et al. [45]	Type 1	0.075%	PBS	30 minutes
Al-Saqi et al. [46]	Type 2	0.1%	Unspecified	45 minutes
Koellensperger et al. [47]	Type 1	0.15%	BSA	45 minutes
Najar et al. [48]	Type 1	0.1%	BSA	45 minutes
Cervelli et al. [49]	Type 1	0.1%	Unspecified	60 minutes
Wu et al. [50]	Type 1	0.1%	DMEM	90 minutes
Yang et al. [51]	Type 1	0.1%	PBS	60 minutes
Yu et al. [52]	Type 1	0.1%	DMEM	60 minutes
Tan et al. [53]	Type 2	1.0%	HBSS/BSA	50 minutes
Kilroy et al. [54]	Type 1	0.1%	PBS/BSA	60 minutes
Jeon et al. [55]	Type 1	0.1%	HBSS/BSA	60 minutes
Rodriguez et al. [39]	Unspecified	0.2%	DMEM/BSA	10 minutes
Devireddy et al. [56]	Type 1	0.1%	PBS/BSA	60 minutes

**Table 2:** Mesenchymal stem cell differentiation testing. COL: collagen; OCN: osteocalcin; ALP: alkaline phosphatase; PPARG: peroxisome proliferator-activated receptor gamma; C/EBP: CCAAT/enhancer-binding protein; ACAN: aggrecan.

Lineage	Histological staining	qPCR gene expression
Osteoblasts	Alizarin Red, Von Kossa	COL 1A1, OCN, runx 2, ALP
Adipocytes	Oil Red O	PPARG 1 and 2, C/EBP a and d
Chondroblasts	Alcian blue	COL 2A1, SOX-9, ACAN

## Abbreviations

MSCs: Mesenchymal stem cells
ADMSCs: Adipose-derived mesenchymal stem cells
IFP: Infrapatellar fat pad
BM: Bone marrow
SVF: Stromal vascular fraction
APC: Articular progenitor cells.
